# High-Performance Polarization Microscopy Reveals Structural Remodeling in Rat Calcaneal Tendons Cultivated In Vitro

**DOI:** 10.3390/cells12040566

**Published:** 2023-02-10

**Authors:** Eli Heber Martins dos Anjos, Maria Luiza Silveira Mello, Benedicto de Campos Vidal

**Affiliations:** Department of Structural and Functional Biology, Institute of Biology, University of Campinas (Unicamp), Campinas 13083-862, SP, Brazil

**Keywords:** tendon explants, tissue culture, polarization microscopy, optical anisotropy

## Abstract

Collagenous tissues exhibit anisotropic optical properties such as birefringence and linear dichroism (LD) as a result of their structurally oriented supraorganization from the nanometer level to the collagen bundle scale. Changes in macromolecular order and in aggregational states can be evaluated in tendon collagen bundles using polarization microscopy. Because there are no reports on the status of the macromolecular organization in tendon explants, the objective of this work was to evaluate the birefringence and LD characteristics of collagen bundles in rat calcaneal tendons cultivated in vitro on substrates that differ in their mechanical stiffness (plastic vs. glass) while accompanying the expected occurrence of cell migration from these structures. Tendon explants from adult male Wistar rats were cultivated for 8 and 12 days on borosilicate glass coverslips (*n* = 3) and on nonpyrogenic polystyrene plastic dishes (*n* = 4) and were compared with tendons not cultivated in vitro (*n* = 3). Birefringence was investigated in unstained tendon sections using high-performance polarization microscopy and image analysis. LD was studied under polarized light in tendon sections stained with the dichroic dyes Ponceau SS and toluidine blue at pH 4.0 to evaluate the orientation of proteins and acid glycosaminoglycans (GAG) macromolecules, respectively. Structural remodeling characterized by the reduction in the macromolecular orientation, aggregation and alignment of collagen bundles, based on decreased average gray values concerned with birefringence intensity, LD and morphological changes, was detected especially in the tendon explants cultivated on the plastic substrate. These changes may have facilitated cell migration from the lateral regions of the explants to the substrates, an event that was observed earlier and more intensely upon tissue cultivation on the plastic substrate. The axial alignment of the migrating cells relative to the explant, which occurred with increased cultivation times, may be due to the mechanosensitive nature of the tenocytes. Collagen fibers possibly played a role as a signal source to cells, a hypothesis that requires further investigation, including studies on the dynamics of cell membrane receptors and cytoskeletal organization, and collagen shearing electrical properties.

## 1. Introduction

Tendons are a specialized group of dense connective tissue that anchors muscles to bones, provides stability, and transmits muscle forces to bones and absorbs impacts, thus enabling the various extension, flexion, and rotation movements of joints [1,2,3,4,5]. The performance of such functions is possible only because of the molecular composition and structural organization of tendons, which endow them with high mechanical resistance to tension and compression and even certain amounts of elasticity [2,5]. The molecular order exhibited by tendon superstructures is a requirement for the development of piezoelectric and pyroelectric properties, in addition to electric transmission potentials [6,7,8].

In terms of composition, tendons are complex structures that are rich in extracellular matrix (ECM) and water that corresponds to 55–70% of the tissue mass [9]. The ECM is mainly formed by collagen molecules that are aligned helically along the tendon axis, nonfibrillar collagens, non-collagen proteins, acid glycosaminoglycans (GAGs) and stromal cells [4]. Because of the ordered organization of their proteins and GAG macromolecules, tendons exhibit important anisotropic optical properties such as birefringence and linear dichroism [8,10,11,12,13,14,15].

Birefringence brightness levels revealed by macromolecularly oriented biological structures that were examined using a polarizing microscope are the result of the sum of intrinsic or crystalline birefringence (Bi) and form or textural birefringence (Bf) phenomena. Bi is defined by Bennett [16] as “anisotropy resulting from the asymmetrical alignment of chemical bonds, ions or molecules within the transmission medium”. It results from interactions of electromagnetic waves that penetrate the transmission medium with electrons and depends on the polarizability, orientation and oscillation strength of all transitions. Bf is the optical anisotropy that is exhibited by “mixed bodies in which asymmetric particles of a given refractive index are dispersed with a preferential orientation in a medium of different refraction index” [8], and it is necessary that the molecular dimensions are small relative to the wavelength of light. The Bf properties of rat tendons have been extensively studied over the years and indicate that the non-collagenous components of the ECM, such as acid GAG polysaccharide chains, also play a role in the macromolecular orientations of these structures [11,12,13,14,15,17].

Among the types of light microscopy, polarization microscopy has thus been extensively used to investigate the submicroscopic structural characteristics and orientation of molecular components of cells or tissues [16]. Even if one intends to study the oriented arrangement of chemical groups in a substrate using infrared microspectroscopy (FTIR), as in the case of tendon collagen fibers, polarized light is required and a polarizing microscope is usually connected to the infrared device [18].

High-performance polarization microscopes are built with every optical component free of strain, permitting that any kind of optical compensator (Sénarmont’s, Bräce-Köhler’s λ/10, λ/20, or λ/30, Ehringhaus’, etc.) be used, Wollaston prisms be inserted, differential interference microscopy be undertaken, and image analysis procedures be performed [15].

Linear dichroism (LD) is the selective absorption of polarized light that results from the molecular orientation of the analyzed substrate relative to the axis of the incident light. It can be expressed mathematically as LD = A_ǁ_ − A_⊥_, where A_ǁ_ and A_⊥_ are the light absorptions exhibited when the long fiber axes of oriented molecular structures are arranged parallel and perpendicular, respectively, to the plane of incidence of polarized light. Because LD in visible light is not usual in biological objects, to study the orientations of collagen and GAG components of collagen fibers using LD properties, it is necessary to stain them with dichroic dyes that have an affinity for proteins (e.g., Xylidine Ponceau 2R, Ponceau SS) [19,20] or GAGs (e.g., toluidine blue, TB) [10,21]. Depending on the molecular orientation of available binding sites in the substrate, the dye molecules become oriented sufficiently to endow the stained substrate with dichroic properties [16].

Birefringence and linear dichroism studies have contributed to determining the changes in the macromolecular organizations of tendon collagen bundles during tendon repair [2,22,23,24,25,26,27,28,29], with advancing aging [30], with animal development [14], and after functional stimulation in response to various biomechanical demands [31]. Statistical determinations of the higher molecular orders and variations in the state of aggregation of collagenous tissues that are performed using polarized light microscopy principles have also enabled describing the oriented supramolecular arrangements of collagen fibers and collagen bundles along the endochondral ossification [32] and in the pericardium [33], chordae tendineae [34], skin [35,36], and enthesis [37].

Tendon explants have been recognized as a valuable tool to investigate mechanisms associated with tendon homeostasis and pathophysiology [4] and as scaffolds after decellularization [38]. Decellularized tendon scaffolds are especially relevant for clinical purposes [38]. However, from the point of view of providing basic scientific knowledge, there are no data reporting the status of macromolecular organizations of collagen bundles in tendon explants during tissue cultivation. This information could be obtained by investigating the optical anisotropic characteristics of collagen fibers in tendons cultivated in vitro that accompany the expected occurrence of cell migration from these structures.

In addition to their structural functions, tendon collagen bundles interact with cells in highly regulated processes, allowing them to respond to stimuli in specific ways by acting as cellular signal transducers [39,40,41,42]. The physico-chemical stimuli generated in tendons due to the presence of different agents in their microenvironments or by mechanical stimuli generated as a result of overload or other mechanical stimulations are supposed to be transmitted through the collagen structure and recognized by cellular components. These events allow cellular responses, enable ECM homeostasis control and initiate various cellular processes, including migration, proliferation, and differentiation. Mechanical interactions between cells and ECM may have dramatic effects on tissue architecture [2,5,8,39,40,43].

Tenocytes are specialized mechanosensitive cells that are organized in interconnected networks and are surrounded by small amounts of specialized matrix with their large cytoplasmic extensions connecting adjacent cells along tendons [44]. These cells play an important role in the production, organization, and maintenance of ECM molecules within the organism, regulate tissue resistance and resilience, interact mechanically and chemically with the ECM, and are involved in the production of matrix-degrading enzymes in tendinopathies [1,3,42,44,45]. Collagen is relevant for the biomechanical functions performed by tenocytes, considering their mechanosensitive nature and dependence on stimuli for correct functioning.

The object of this work, by using optical anisotropy methods, was to investigate how the macromolecularly oriented organization of adult tendon collagen bundles responds to tissue cultivation in vitro and whether it is affected by explant adhesion substrates with different states of mechanical stiffness (plastic vs. glass). This investigation also aimed to detect whether cell migration in tendons cultivated in vitro is associated with changes in the oriented organizations of tendon fibers and responds differentially under tissue cultivation on glass and plastic substrates. Considering that the methodology used is sufficiently sensitive to detect the submicroscopic structure and orientation of the macromolecular components of the tendon collagen fibers, the results obtained in this work will potentialize further studies on tendon explants obtained from newborn and aged animals where the organization and composition of the collagen bundles and the cellularity abundance are known to differ in vivo [14,30]. Tendons other than the calcaneal type could also be scrutinized for comparative purposes. Whether results depending on the substrate surface here proposed for explant adhesion differ, selection of one of them for further experiments adding functionalized elements could be undertaken not only regarding comparison of the organization of the tendon collagen bundles but also regarding behavior of cell migration.

## 2. Materials and Methods

### 2.1. Animals and Sample Preparations

Adult male Wistar rats (*Rattus norvegicus albinus*) (*n* = 5) were provided by the Multidisciplinary Center of Biological Investigation (CEMIB) at the University of Campinas (Campinas, Brazil). The animals were reared under standard conditions, given water ad libitum and fed extruded chow (Nuvital^®^, Colombo, Brazil). Animal care and use protocols were approved by the Ethics Committee for Animal Use at the University of Campinas (registration no. 5064-1/2018) and followed the guidelines established by the Brazilian National Council for the Control of Animal Experimentation (CONCEA).

Calcaneal tendons isolated from these rats were cultivated under sterile conditions on 24 mm square colorless borosilicate glass coverslips (*cov*) (*n* = 3) or placed in direct contact with the surfaces of cultures of 35 mm ø nonpyrogenic polystyrene plastic dishes (*pla*) (*n* = 4) for 8 and 12 days in DMEM/HAMF12 (*v*/*v*) (DMEM, Sigma-Aldrich^®^, St. Louis, MO, USA; HAMF12, Vitrocell Embriolife, Campinas, Brazil) medium supplemented with 10% bovine fetal serum and penicillin/streptomycin (Sigma-Aldrich^®^, 100 IU/mL and 100 µg/mL) at 37 °C, 95% humidity and 5% CO_2_. The media were changed every 72 h. Tissues were maintained in 4 mL of medium per dish. Tendons not cultivated in vitro were used as controls (*n* = 3). To improve the adhesion of the tendon explants of the *pla* group to the dish surfaces, they were incubated in an absence of medium for 60 min in a cell culture oven at 37 °C prior to beginning the proper culture procedures. All tendon explants were maintained without no longitudinal or compressive forces applied to the tissues.

Tendons were fixed in 4% paraformaldehyde, embedded in Histosec (Merck, Darmstadt, Germany), and cut to a thickness of 8 µm with a Microm HM 315 microtome (Waldorf, Germany).

### 2.2. Birefringence

Birefringence analysis was performed in tendon sections that were immersed in deionized water (Direct-Q3, Molsheim, France), and the maximum brilliance intensity corresponds to the sum of the Bi and Bf phenomena [11,12,15]. Determination of birefringence brilliances was performed using an Olympus BMX-51 high-performance polarizing microscope (Olympus America Center, Center Valley, PA, USA) equipped with a Q-color 5 camera (Olympus America Center), differential interference contrast optics (DIC-PLM), and Sénarmont compensator. Monochromatic light with λ = 540 nm was used. Image-Pro Plus software, v.6.3 for Windows™ (Media Cybernetics, Inc., Silver Spring, MD, USA) was used to measure the birefringence brilliance intensities as average gray values (GA) that, in the range of 25 to ~250 pixels, were linearly correlated with optical retardations, as previously described [15]. Magnification calibration was performed considering a pixel/µm correlation = 7.050 for the 40× objective. The birefringence brightness levels were associated with the linear amplitudes of pixel values such that the extreme 255-pixel value was associated with the maximum brightness, whereas pixels with 0 values represented an absence of brightness. The section thickness used meant that the collagen fiber concentrations reflected the variations in fiber orientation with respect to the long axes of tendons and their packing degrees, which thus enabled an accurate birefringence analysis.

Because the birefringence brilliance intensities observed in tendon sections were distributed unevenly along the tendon axes and large areas of images had to be analyzed using image analysis, the total area of images obtained after birefringence compensation was segmented into smaller subareas (Supplementary Figure S1). The software was used to collect the gray values obtained individually into integrated gray values. The image areas that were covered for measurement were 10^5^ µm^2^; the number of average gray measurements was between 206 and 297, which was sufficient to cover the possible variations in anisotropy.

### 2.3. Differential Interference Contrast Microscopy (DIC-PLM)

A polarizing microscope (Olympus BMX-51) equipped with two Wollaston prisms was used for DIC-PLM analysis. Optical path differences were detected by displacing the prism positioned under the analyzer and comparing the resulting interference colors with those on the Michel-Lévy color scale [37].

### 2.4. Linear Dichroism (LD)

Sections 8 µm thick from tendons fixed in 4% paraformaldehyde were stained for 3 min with a 0.025% solution of Ponceau SS (Aldrich, Milwaukee, Brookfield, WI, USA) in 3% acetic acid prepared in bidistilled water, followed by rinsing in 3% acetic acid for 2 min and then placed in distilled water to investigate the LD properties of tendon proteins [20]. Ponceau SS is a sulfonic azo-dye that attaches electrostatically to protein -NH^3+^ groups. If the protein -NH³^+^ groups available to dye binding occur in the substrate with an oriented distribution as in the case of collagenic proteins, the stained substrate will exhibit linear dichroism because the dye molecules attached to the substrate will acquire an ordered stereo arrangement and the oriented dye molecules are susceptible to exhibit a selective absorption of polarized light [20]. The intensity of this optical phenomenon revealed using polarization microscopy will depend on the level of molecular orientation of the substrate [20].

Sections taken from tendons were also fixed in paraformaldehyde and stained with a 0.025% TB (Merck) solution in McIlvaine buffer at pH 4.0 for 15 min and then rinsed in McIlvaine buffer at pH 4.0 and in a stabilizer solution of 4% ammonium molybdate aqueous solution for 4 min to investigate LD in tendon GAGs [13]. The pH of the staining solution enabled the identification of which binding sites of the stained substrate were available for electrostatic binding of the dye molecules [21]. TB is a thiazine cationic dye that in solution at pH 4.0 attaches electrostatically to -SO³^−^ and COO^−^ groups like those present in GAGs. If the available binding sites in the stained substrate are positioned with an oriented distribution, the stereoarrangement of the dye molecules will also be oriented and the phenomenon of linear dichroism will be exhibited using polarization microscopy, thus revealing the status of molecular orientation of the stained substrate [10,13,14,21].

The stained preparations were examined under polarized light using an AxioPhot microscope (Carl Zeiss, Oberkochen, Germany) (Ponceau SS-stained sections) or using an Olympus BMX-51 high-performance polarization microscope (TB-stained sections), both equipped with 40× objectives. The long axis of the tendons was positioned parallel (A_ǁ_) and perpendicular (A_⊥_) to the plane of the PPL.

### 2.5. Tenocyte Migration

The tenocyte distributions were determined during a 12-day period in tendons cultivated in vitro on plastic and glass surfaces. Examinations were performed using an Olympus IX50 inverted microscope (Olympus America Center), and images were captured using Motic Image Plus 2.0 software (Motic Asia, Kowloon, Hong Kong).

### 2.6. Statistics

Calculations and statistical analyses were performed using Minitab 14^®^ software (State College, PA, USA). Kruskal–Wallis and Mann–Whitney tests were used to assess statistical significance when comparing GA values. *p* < 0.05 was considered the critical level to reject the null hypothesis.

## 3. Results

### 3.1. Birefringence

**Figure 1:**
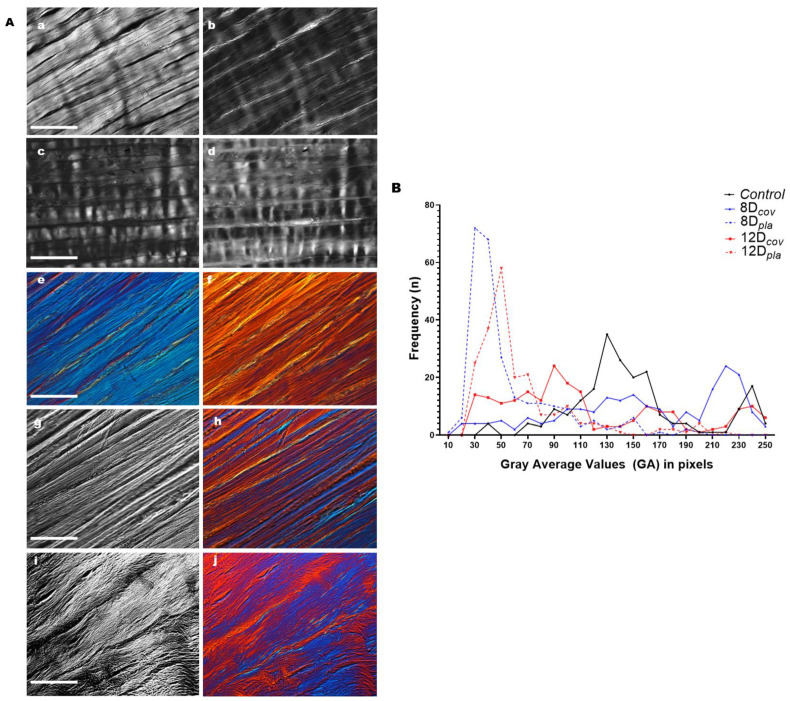
Birefringence images and respective average gray evaluations obtained for sections of control and cultivated rat calcaneal tendons. (**A**). Images representative of control tendon sections are shown before (**a**,**c**,**e**) and after (**b**,**d**,**f**) birefringence compensation. The intensity variations in birefringence brilliance levels are caused by variations in collagen fiber orientation with respect to the crossed polarizers. When the long axes of tendons were positioned parallel to the polarizer’s PPL, the crimp structure becomes identifiable (**c**,**d**). Interference colors obtained when using the DIC-PLM system are depicted for sections of control tendons (**e**,**f**) and for tendons cultivated on glass coverslips (**h**,**j**). Images (**h**,**j**), obtained with the DIC-PLM system, correspond to images g and i, respectively, which were obtained with ordinary polarization microscopy. Structural changes in collagen bundles were visualized with increasing time of tissue cultivation (**g**,**h**—8 days; **i**,**j**—12 days). Scale bars equal 50 µm. (**B**). Polydispersed distributions of birefringence average gray values expressed in pixels corresponding to optical retardations for collagen bundles of tendon sections immersed in water. The higher frequencies of average gray values that were detected in control tendons shift to smaller values in tendons cultivated for 8 and 12 days on plastic dishes and in tendons cultivated for 12 days on glass coverslips. The long axes of the tendons were positioned at 45° with respect to the PPL.

Typical birefringence brilliance intensities were detected when the collagen bundles of the control (Figure 1A(a–f)) and cultivated tendons (Figure 1A(g–j)) were examined using polarization microscopy. The maximum birefringence brilliance in each case was observed when the collagen fibers were positioned at 45° in relation to the crossed polarization planes of the polarizer and analyzer (Figure 1A(a,g,i)). Birefringence compensation of the images shown in Figure 1A(a,c,e) is visualized in Figure 1A(b,d,f), respectively. When the main axes of the collagen bundles were positioned parallel to the azimuths of the polarizers, the birefringence brilliances related to the crimp structure became evident (Figure 1A(c)). The second-order blue interference color exhibited by control tendon collagen fibers using the DIC-PLM system (Figure 1A(e)) changes into a second-order red interference color after birefringence compensation (Figure 1A(f)). These birefringence interference colors are due to optical path differences between the trajectories of the rays emerging from the material as a result of the molecular orientation of the structural arrangement of the collagen fibers.

Heterogeneity in the birefringence brilliance distributions in tendon collagen bundles, especially under in vitro cultivation, was confirmed by the variations in interference colors (Figure 1A(h,j)) and by the distributions of the birefringence GA values along the collagen bundles of the tendon structures (Table 1, Figure 1B). Unpackaging of collagen bundles was especially evident in tendon fragments that were cultivated for 12 days (Figure 1A(i,j)).

A polydispersed distribution of the GA values in pixels corresponding to birefringence optical retardation along tendon structures, as a result of variations in the structural organization levels of the collagen bundles, was detected, especially under culture conditions (Figure 1B). Higher frequencies of GA values in the control tendons were found in the range of 110–160 pixels and shifted to ~30–50 pixels in tendons cultivated for 8 and 12 days on plastic dishes and to 30–110 pixels in tendons cultivated for 12 days on glass coverslips (Figure 1B). This frequency polydispersion reflected the large standard deviation of the GA values in the cultivated tendons (Table 1) and is a result of structural remodeling of the tendon collagen bundles.

The GA values among collagen bundles were compared under the culture conditions used and with respect to the control, and differences that were highly significant in most cases were observed (Table 2). Raw GA dataset is deposited in the repository of the University of Campinas [46].

### 3.2. Linear Dichroism (LD)

**Figure 2:**
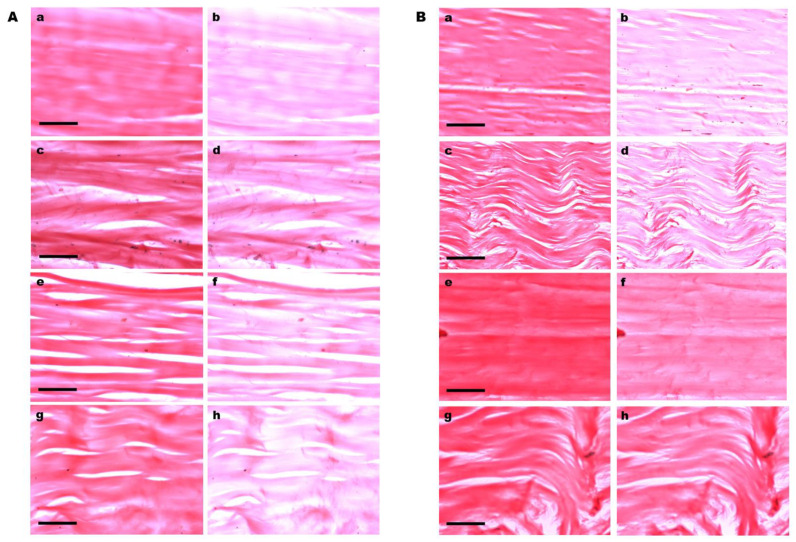
Positive LD in Ponceau SS-stained tendon collagen bundles. (**A**) Images representative of stained collagen bundles in control tendons (**a**,**b**) and tendons cultivated on glass coverslips (**c**–**h**). The long axes of the tendons were positioned parallel (**a**,**c**,**e**,**g**) and perpendicular (**b**,**d**,**f**,**h**) to the PPL. Images (**g**,**h**) correspond to the extremity tendon zone distal to the region originally occupied by the enthesis in vivo. (**B**) Images representative of stained collagen bundles in tendons cultivated on plastic dishes (**a**–**h**). The long axes of the tendons were positioned parallel (**a**,**c**,**e**,**g**) and perpendicular (**b**,**d**,**f**,**h**) to the PPL. Images (**c**,**d**,**g**,**h**) were obtained at zones distal to the region originally occupied by the enthesis. A less intense LD phenomenon was observed in tendons cultivated for 12 days, especially at zones distal to the region originally occupied by the enthesis (**g**,**h**). Scale bars equal 50 µm (**A**(**a**–**h**)) and (**B**(**e**–**h**)) and 100 µm (**B**(**a**–**d**)).

LD phenomena with positive signs (A_ǁ_ > A_⊥_) were intense in Ponceau SS-stained collagen fibers of the control tendons (Figure 2A(a,b)) and in tendons cultivated for 8 days on plastic substrate (Figure 2B(a,b)), which persisted maintained in varying manners along tendons cultivated on glass coverslips (Figure 2A(c–h)) and were less evident in tendons cultivated for 12 days on plastic dishes (Figure 2B(e–h)), especially in the region of the tendons distal to the region originally occupied by the enthesis in vivo (Figure 2B(g,h)). It is worth mentioning that enthesis, properly, was not included in the explants used in this study.

**Figure 3:**
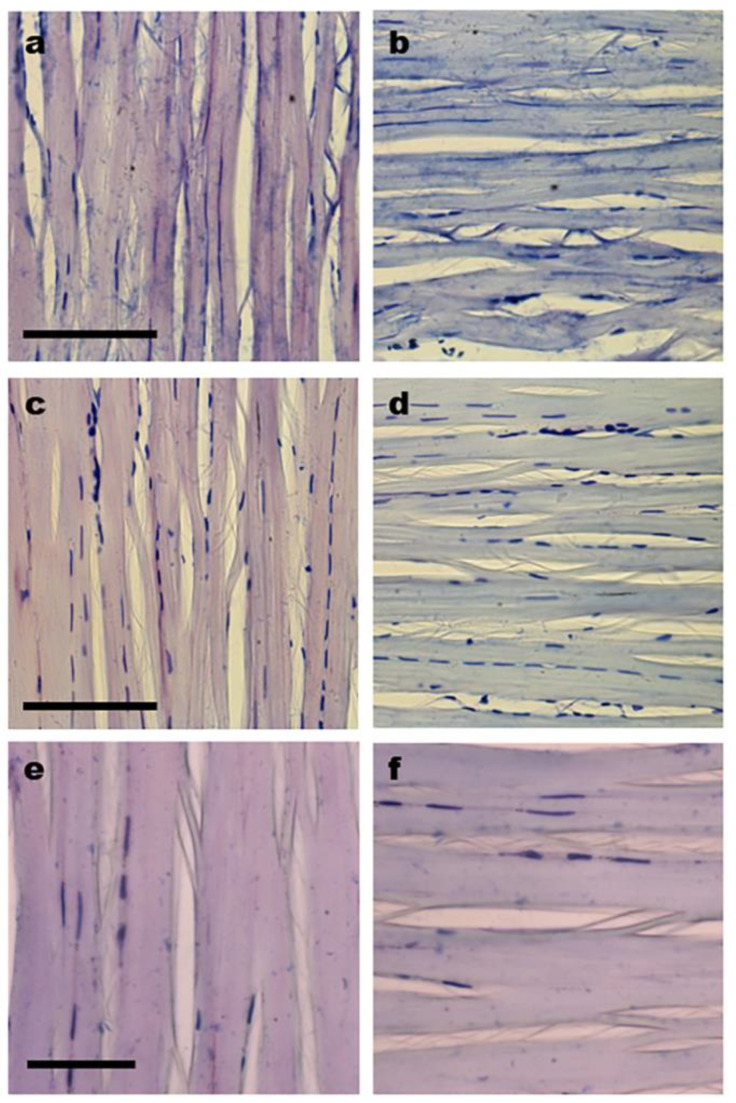
Negative LD in TB-stained tendon collagen bundles. Images representative of stained control tendons (**a**,**b**) and tendons cultivated for 8 (**c**,**d**) and 12 (**e**,**f**) days on glass coverslips (**c**–**f**). The long axes of the tendons were positioned perpendicular (**a**,**c**,**e**) (violet color) and parallel (**b**,**d**,**f**) (blue color) to the PPL. Images e and f were obtained for zones distal to the region originally occupied by the enthesis in vivo. The LD phenomenon is maintained in cultivated tendons, although it exhibits slightly less intense colors in comparison with control tendons. Scale bars equal 50 µm.

Typical LD phenomena with negative signs (A_⊥_ > A_ǁ_) were detected in TB-stained collagen bundles of the control and cultivated tendons, although less intense colors were observed in the cultivated tissues (Figure 3a–f). Different colors were exhibited by the collagen bundles when they were positioned parallel (blue) and perpendicular (violet) to the PPL (Figure 3a–f).

### 3.3. Cell Distribution

**Figure 4:**
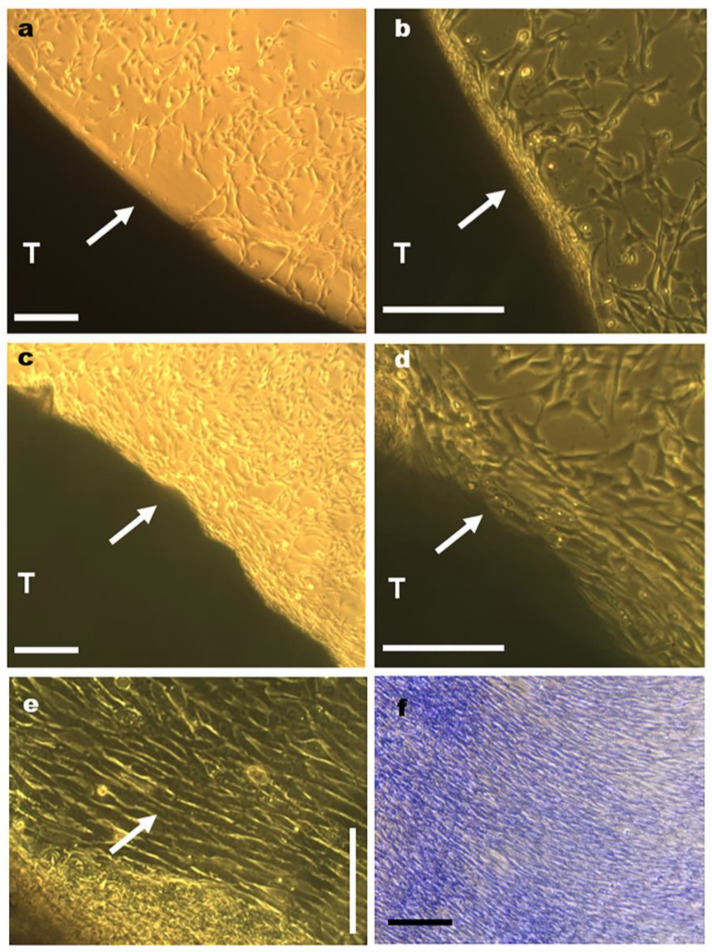
Tenocyte distributions in tendons (T) cultivated in vitro as observed with inverted light microscope. Images representative of the distribution of tenocytes that migrated from the lateral regions of tendons (**a**–**d**, arrows) to the plastic substrate after a 6-day period of tendon cultivation (**a**,**b**) and that acquired a preferential orientation parallel to the long tendon axis 24 h later (**c**–**e**). Details of dense tenocyte distributions oriented parallel to the long axis of the tendon are indicated by an arrow (**e**). The number of the parallel aligned rows after a 12-day period of tendon cultivation increased even more as seen in a preparation stained with toluidine blue at pH 4.0 (**f**). Scale bars equal 250 µm (**a**–**d**,**f**) and 100 µm (**e**).

While tenocyte migration was evident immediately after a 5-day culture period in tendons cultivated on plastic dishes (Figure 4a,b), migration was detected only after a 12-day culture period in tendons cultivated on glass surfaces, and in this case, it occurred with low cell frequencies.

Cell migration occurred primarily from the lateral zones of tendons (Figure 4a–e; Supplementary Figure S2a,d), although greater amounts of collagen bundle unpackaging were observed in fragmentation zones that were distal to the region originally occupied by the enthesis, where lower levels of cell migration occurred (Supplementary Figure S2c).

Initially, cells that migrated to the surfaces of the plastic dishes appeared to be distributed without a preferential direction (Figure 4a,b). With increasing culture time from a 6- to a 7-day period, cell proliferation, and migration of cells toward the surfaces of the dishes, the tenocytes became aligned in parallel rows with respect to the long axis of tendons (Figure 4c–e). After a 12-day culture period, the number of the aligned parallel rows increased even more occupying a distance of ~1000 µm from the lateral boundary of the explant (Figure 4f).

## 4. Discussion

The optical anisotropic birefringence and LD results obtained demonstrated structural remodeling that was characterized by changes in macromolecular orientations and aggregational states of the collagen bundles in rat tendons subjected to tissue culture for 8 and 12 days, especially upon cultivation on a plastic substrate. The occurrence of the optical anisotropic phenomenon of birefringence has been widely reported for tendon collagen bundles based on visual observations or quantitatively evaluated through measurements of optical retardations [2,8]. Using image analysis procedures, birefringence brilliances can also be evaluated in tendon collagen bundles through determinations of average gray (GA) values that correspond to optical retardations [15]. The decreased GA values detected in rat tendons cultivated in vitro indicate reductions in the crystallinity levels of tendon suprastructures due to the lower aggregation states of their molecular components. While the LD findings obtained from the Ponceau SS-stained preparations support this idea for collagen and other structural proteins of tendons [20], preparations stained with TB solution at pH 4.0 indicated subtle changes in the macromolecular orientation of the GAG components of tendons [21].

Determinations of optical anisotropy properties have already enabled the detection of changes in the stereo arrangement of the macromolecular components of collagen bundles in tendons under different physiological states, such as in detached or surgically implanted tendons of *Cavia porcellus* L. [22], during repair after rat tendons were surgically removed [23,25,28], in rat tendons with aging [30], in rat tendons after denervation followed by different mechanical stimuli promoted by physical exercise [31], after tendon immobilization followed by stretching [26], and in the enthesis [37]. Valdetaro et al. [47], who used polarized light microscopy and image analysis, also identified structural changes in the fibrillar components of the ECM of the human amniotic membrane and rabbit limbic stroma after explant culture. Birefringence and LD evaluation and DIC-PLM analysis of transected rat tendons that were treated with adipose-derived mesenchymal stem cells (ADMSC) or GDF-5, or both, demonstrated an increased expression of certain genes related to tissue remodeling, that contributed to a greater organization of collagen fibers in the ADMSC-treated samples; treatment of injured tendons with ADMSC was more effective when compared to treatment with an ADMSC-GDF-5 association [28].

Updated studies reinforce the applicability of polarization microscopy in determining molecular order in tendon structures. The evaluation of birefringence expressed as average gray values has allowed the detection of improvement in the state of aggregation and organization of tendon collagen bundles in a study using low-level laser and adipose-derived stem cells [48]. Birefringence and LD studies carried out using a biodegradable new fibrin sealant derived from a spider venom associated with adipose-derived stem cells on rat tendon injuries allowed determining changes in the macromolecular organization of collagen bundles during tendon repair [29].

An advantage of the present detection of changes in birefringence intensity using image analysis was that the uneven distribution of the gray values corresponding to compensated birefringence across the tendon section could be evaluated quickly and across a large image area of the preparations. The use of the polarization microscopy methods described in this study has a limitation which is a requirement of experimental expertise from the microscopist. The added value of these methods is that images containing informative algorithms fundamental for the interpretation of the results can be obtained.

Although there were reductions in the birefringence GA values of collagen bundles and decreased LD values for the Ponceau SS-reactive proteins in tendons cultivated directly on glass coverslips or plastic dishes, tissue remodeling was more visually evident, and cell migration occurred earlier and more intensely in samples that were incubated in an oven and then cultivated on the surfaces of plastic dishes. Substrate stiffness differences upon which tendon cultivations occurred thus differentially affected the rate of the altered stereo arrangement process of tendon structures and permitted cell motility and migration toward the culture adhesion substrate with increasing culture time [49]. The decreased alignment of collagen bundles, especially at the 12-day period of tendon cultivation on plastic dishes, was probably induced by the deprivation of mechanical stimulation that the tendons experienced under in vitro conditions [50,51] and because the culture substrates were not functionalized, for instance, with type I collagen [51].

Preferential migration of tenocytes to lateral regions of rat tendon explants is consistent with reports showing that cells originating from the surfaces of equine digital flexor tendon explants migrated faster and exhibited higher replication rates than cells originating from the inner regions of these tendons [52]. Migration of cells from the center to the margin zone in a rabbit-derived explant of the anterior cruciate ligament after 20 days of culturing has also been reported [53]. The fact that cells that initially migrated to the substrate surface assumed random distributions with respect to the long axes of tendons and with advancing time became oriented parallel to this axis, which caused all of the migrating tenocytes to assume this orientation, suggests that as mechanosensitive cells, tenocytes recognize the orientation of the suprastructure of the collagen bundles and position themselves parallel to them. Because biochemical signals translated by tenocytes are related to the induction of cellular functions and may increase tissue maturation, remodeling, and/or repair [54,55], the present study suggests that tenocytes sense the biomechanical signals arising from structural tissue rearrangements in culture, which involve decreases in the aggregation state and degree of crystallinity of the suprastructure and respond by translating such biomechanical signals into biochemical signals capable of regulating the migration process and influencing ECM remodeling. Cell alignment patterns that are axial to explants thus may indicate the role of collagen as a signal source for cells, acting as a transducer, which are able to guide even cells that have already migrated to the surface of the plaque. This is a hypothesis that requires further investigation, including studies on the dynamics of cell membrane receptors and cytoskeletal organization, and collagen shearing electrical properties.

Mechanical interactions between cells and collagen fibers are known to affect tissue architecture [5,43]. According to Garcia and Garcia [49], cells have the ability to sense changes in the supraorganization, biochemical properties, and rigidity of the ECM and respond to these signals by activating various mechano-transduction signaling pathways that result in ECM remodeling. In the present study, the stimuli induced in rat tendons by in vitro culture conditions resulted in adaptive responses to new demands that caused the collagen fibers to a certain shrinkage, and this, in turn, apparently induced cell migration. The period during which tenocyte migration occurred from rat tendon explants did not differ much from the period during which human finger tendon explant tenocytes begin migrating and adhering to culture flasks (7–14 days) [56,57].

The cell migration phenomenon in rat tendons cultivated in vitro demonstrates the performance of the structural components of tendons, with special emphasis on collagen, in its role as a transducer of electrical signals and potentials. As previously reported, the superstructures of collagen bundles endow them with anisotropic, piezoelectric, and pyroelectric optical properties, in addition to electric transmission potentials, that extend from molecular to nanometric organizational levels. Chirality, rotational symmetry, and liquid crystal twisted grain boundaries are necessary conditions for the occurrence of such properties [39]. Mechano-transduction is an important mechanism through which some mechanical stresses act on cells and initiate specific intracellular signals that promote cell growth and survival, manage the morphology, architecture, and maintenance of superstructures, and affect various metabolic responses [58,59,60,61,62,63].

There are reports describing increased numbers of tenocytes in lesion areas during late-stage healing in rat tendons, where these cells proliferate and become responsible for the abundant deposition of ECM [64,65,66]. Interestingly, although we observed certain numbers of cells at the proximal and distal zones of the explanted tendons, reduced cell migration was detected at these regions, possibly because of their minor degree of compaction that could influence how cells perceive mechano-transduction signals that emanate from collagen bundles. 

## 5. Conclusions

**Figure 5:**
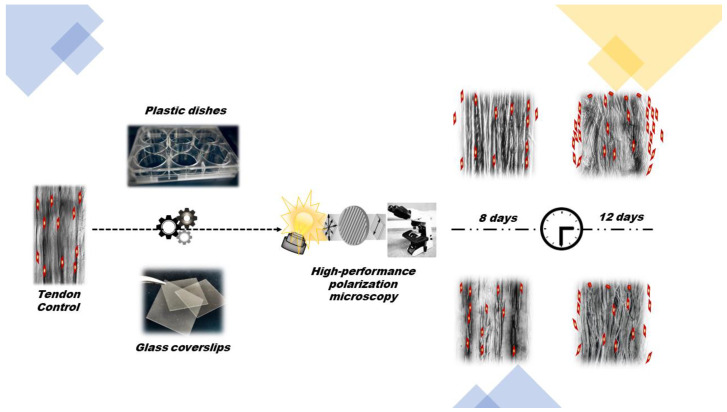
Graphical representation summarizing the main results of the present study.

Structural remodeling involving reductions in crystallinity levels, which were evaluated based on the changes in optical anisotropic characteristics such as birefringence and linear dichroism occurs in rat calcaneal tendon explants cultivated in vitro for 8 and 12 days on plastic and glass substrates. These changes affect the aggregation states and macromolecular orientations of components in tendon collagen bundles, such as collagen and non-collagen structural glycoproteins and GAG, and are accompanied by tenocyte migration to culture substrates. Glass substrates appear to delay the structural rearrangement process of collagen fibers and of tenocyte migration, possibly because of their stiffness. The plastic substrate used for calcaneal tendon explant adhesion reveals more suitable than glass to detect changes in the stereo arrangement of collagen bundles and facilitation of cell migration during tissue culture. Cell migration and alignment in rows parallel to the long axes of the tendons cultivated on plastic substrates are possibly due to the mechanosensitive nature of tenocytes. Further studies involving digestion of GAG components by specific enzymes would certainly reveal the level of participation of these macromolecules on the changes in stereo arrangement of collagen bundles and cell migration detected during tendon explant culture. Figure 5 graphically summarizes the results of the present study.

## Figures and Tables

**Table 1:** Frequency distributions of birefringence average gray values corresponding to optical retardations in sections of rat calcaneal tendons cultivated in vitro.

Items	Average Gray Values in Pixels (GA)				
	Control	8D*cov*	8D*pla*	12D*cov*	12D*pla*
No. of measurements	206	206	251	206	207
Minimum GA	40.28	22.57	14.15	27.26	28.90
Maximum GA	247.91	247.90	199.50	247.00	218.40
Median	139.95	157.57	41.21	96.60	52.84
Mean	148.32	158.27	54.86	114.98	64.96
Standard Deviation	46.57	61.92	34.56	64.62	36.55

D, days; *cov*, cultivation on glass coverslips; *pla*, cultivation on plastic dishes.

**Table 2:** Statistical comparison of birefringence average gray values (GA) between collagen bundles in rat tendons differently cultivated in vitro.

Parameter	Comparison	Test	*p*	Decision
GA (pixels)	8Dcov vs. 12Dcov vs. Control	KW	<0.0001	SS
	8Dcov vs. Control	MW	0.0460	S
	12Dcov vs. Control	MW	<0.0001	SS
	8Dcov vs. 12Dcov	MW	<0.0001	SS
	8Dpla vs. 12Dpla vs. Control	KW	<0.0001	SS
	8Dpla vs. Control	MW	<0.0001	SS
	12Dpla vs. Control	MW	<0.0001	SS
	8Dpla vs. 12Dpla	MW	<0.0001	SS
	8Dcov vs. 8Dpla vs. Control	KW	<0.0001	SS
	8Dcov vs. 8Dpla	MW	<0.0001	SS
	8Dcov vs. 12Dpla	MW	<0.0001	SS
	12Dcov vs. 12Dpla vs. Control	KW	<0.0001	SS
	12Dcov vs. 12Dpla	MW	<0.0001	SS
	12Dcov vs. 8Dpla	MW	<0.0001	SS

Present data refer to the n values presented in Table 1 (No. of measurements). *p* < 0.05 was considered the critical level to reject the null hypothesis. The individual GA values composed a dataset that was deposited in a public repository at the University of Campinas [46]. The data represent separate experiments. cov, cultivation on glass coverslips; D, days; KW, Kruskal–Wallis; MW, Mann–Whitney; pla, cultivation on plastic dishes; S, significant; SS, highly significant; vs., versus.

## Data Availability

Raw GA dataset is deposited in the repository of the University of Campinas at https://doi.org/10.25824/redu/7OQWES.

## Supplementary Materials

The following supporting information can be downloaded at: https://www.mdpi.com/article/10.3390/cells12040566/s1, Figure S1: A large area of a tendon section after birefringence compensation; Figure S2: Tenocyte distribution in a rat tendon (T) at its 8-day-culture on plastic surface.

## References

[1] O’Brien M., Maffulli N., Renström P., Leadbetter W.B. (2005). Anatomy of Tendons. Tendon Injuries.

[2] de Aro A.A., Vidal B.D.C., Pimentel E.R. (2012). Biochemical and anisotropical properties of tendons. Micron.

[3] Wang J.H.-C., Thampatty B.P., Verbruggen S. (2008). Advances in Tendon Mechanobiology. Mechanobiology in Health and Disease.

[4] Wunderli S.L., Blache U., Snedeker J.G. (2020). Tendon explant models for physiologically relevant *in vitro* study of tissue biology—A perspective. Connect. Tissue Res..

[5] Izu Y., Adams S.M., Connizzo B.K., Beason D.P., Soslowsky L.J., Koch M., Birk D.E. (2021). Collagen XII mediated cellular and extracellular mechanisms regulate establishment of tendon structure and function. Matrix Biol..

[6] Athenstaedt H. (1974). Pyroelectric and piezoelectric properties of vertebrates. Ann. N. Y. Acad. Sci..

[7] Akagi K., Piao G., Kaneko S., Sakamaki K., Shirakawa H., Kyotani M. (1998). Helical Polyacetylene Synthesized with a Chiral Nematic Reaction Field. Science.

[8] Vidal B. (2010). Form birefringence as applied to biopolymer and inorganic material supraorganization. Biotech. Histochem..

[9] Jaiswal D., Yousman L., Neary M., Fernschild E., Zolnoski B., Katebifar S., Rudraiah S., Mazzocca A.D., Kumbar S.G. (2020). Tendon tissue engineering: Biomechanical considerations. Biomed. Mater..

[10] Vidal B.D.C. (1963). Pleochroism in tendon and its bearing to acid mucopolysaccharides. Protoplasma.

[11] Vidal B.D.C. (1965). The part played by the mucopolysaccharides in the form birefringence of the collagen. Protoplasma.

[12] Vidal B.D.C. (1980). The part played by proteoglycans and structural glycoproteins in the macromolecular orientation of collagen bundles. Cell. Mol. Biol..

[13] Mello M.L., Vidal B.C. (1973). Anisotropic properties of toluidine blue-stained collagen. Ann. Histochim..

[14] Vidal B.C., Mello M.L. (1984). Proteoglycan arrangement in tendon collagen bundles. Cell. Mol. Biol..

[15] Vidal B.C., Mello M.L.S. (2010). Optical anisotropy of collagen fibers of rat calcaneal tendons: An approach to spatially resolved supramolecular organization. Acta Histochem..

[16] Bennet S., Jones R.M. (1967). The Microscopical Investigation of Biological Materials with Polarized Light. McClung’s Handbook of Microscopical Technique.

[17] Vidal B.D.C. (1986). Evaluation of the carbohydrate role in the molecular order of collagen bundles: Microphotometric measurements of textural birefringence. Cell. Mol. Biol..

[18] Vidal B.C. (2013). Using the FT-IR linear dichroism method for molecular order determination of tendon collagen bundles and nylon 6. Acta Histochem..

[19] Vidal B.D.C. (1970). Dichroism in collagen bundles stained with Xylidine-Ponceau 2R. Ann. Histochim..

[20] Vidal B.C., Mello M.L.S. (2005). Supramolecular order following binding of the dichroic birefringent sulfonic dye Ponceau SS to collagen fibers. Biopolymers.

[21] Vidal B.D.C., Mello M.L.S. (2019). Toluidine blue staining for cell and tissue biology applications. Acta Histochem..

[22] Vidal B.D.C. (1966). Macromolecular disorientation in detached tendons. Protoplasma.

[23] Mello M.L.S., Godo C., Vidal B.C., Abujadi J.M. (1975). Changes in macromolecular orientation on collagen fibers during the process of tendon repair in the rat. Ann. Histochim..

[24] da Cunha A., Parizotto N., Vidal B.D.C. (2001). The effect of therapeutic ultrasound on repair of the Achilles tendon (tendo calcaneus) of the rat. Ultrasound Med. Biol..

[25] Carrinho P.M., Renno A.C.M., Koeke P., Salate A.C.B., Parizotto N.A., Vidal B.C. (2006). Comparative Study Using 685-nm and 830-nm Lasers in the Tissue Repair of Tenotomized Tendons in the Mouse. Photomed. Laser Surg..

[26] Aro A.A., Vidal B.C., Tomiosso T.C., Gomes L., Matiello-Rosa S.M.G., Pimentel E.R. (2008). Structural and Biochemical Analysis of the Effect of Immobilization Followed by Stretching on the Calcaneal Tendon of Rats. Connect. Tissue Res..

[27] Aro A., Freitas K., Foglio M., Carvalho J., Dolder H., Gomes L., Vidal B., Pimentel E. (2013). Effect of the Arrabidaea chica extract on collagen fiber organization during healing of partially transected tendon. Life Sci..

[28] De Aro A.A., Carneiro G.D., Teodoro L.F.R., Da Veiga F.C., Ferrucci D.L., Simões G.F., Simões P.W., Alvares L.E., De Oliveira A.L.R., Vicente C.P. (2018). Injured Achilles Tendons Treated with Adipose-Derived Stem Cells Transplantation and GDF-5. Cells.

[29] Frauz K., Teodoro L.F.R., Carneiro G.D., da Veiga F.C., Ferrucci D.L., Bombeiro A.L., Simões P.W., Alvares L.E., de Oliveira A.L.R., Vicente C.P. (2019). Transected Tendon Treated with a New Fibrin Sealant Alone or Associated with Adipose-Derived Stem Cells. Cells.

[30] Mello M.L.S., Vidal B., De Carvalho A., Caseiro-Filho A. (1979). Change with Age of Anisotropic Properties of Collagen Bundles. Gerontology.

[31] Vilarta R., Vidal B.D.C. (1989). Anisotropic and Biomechanical Properties of Tendons Modified by Exercise and Denervation: Aggregation and Macromolecular Order in Collagen Bundles. Matrix.

[32] Vidal B.C. (1977). Acid glycosaminoglycans and endochondral ossification: Microspectrophotometry evaluation and macromolecular orientation. Cell. Mol. Biol..

[33] Braga-Vilela A.S., Pimentel E.R., Marangoni S., Toyama M.H., Vidal B.D.C. (2008). Extracellular Matrix of Porcine Pericardium: Biochemistry and Collagen Architecture. J. Membr. Biol..

[34] Vidal B.D.C., Mello M.L.S. (2009). Structural organization of collagen fibers in chordae tendineae as assessed by optical anisotropic properties and Fast Fourier transform. J. Struct. Biol..

[35] Silva D.D.F.T.D., Vidal B.D.C., Zezell D.M., Zorn T.M.T., Ribeiro M.S., Núñez S.C. (2006). Collagen birefringence in skin repair in response to red polarized-laser therapy. J. Biomed. Opt..

[36] Ribeiro J.F., dos Anjos E.H.M., Mello M.L.S., Vidal B.D.C. (2013). Skin Collagen Fiber Molecular Order: A Pattern of Distributional Fiber Orientation as Assessed by Optical Anisotropy and Image Analysis. PLoS ONE.

[37] Vidal B.C., dos Anjos E.H.M., Mello M.L.S. (2015). Optical anisotropy reveals molecular order in a mouse enthesis. Cell Tissue Res..

[38] Youngstrom D., Barrett J.G. (2016). Engineering Tendon: Scaffolds, Bioreactors, and Models of Regeneration. Stem Cells Int..

[39] Vidal B.C. (1993). Cell and extracellular matrix interaction: A feedback theory based on molecular order and recognition-adhesion events. Rev. Fac. Ciênc. Med. Unicamp.

[40] Vidal B.D.C. (2003). Image analysis of tendon helical superstructure using interference and polarized light microscopy. Micron.

[41] Buschmann J., Bürgisser G.M. (2017). Structure and Function of Tendon and Ligament Tissues. Biomechanics of Tendons and Ligaments.

[42] Vermeulen S., Vasilevich A., Tsiapalis D., Roumans N., Vroemen P., Beijer N.R., Eren A.D., Zeugolis D., de Boer J. (2018). Identification of topographical architectures supporting the phenotype of rat tenocytes. Acta Biomater..

[43] Zhang T., Day J.H., Su X., Guadarrama A.G., Sandbo N.K., Esnault S., Denlinger L.C., Berthier E., Theberge A.B. (2019). Investigating Fibroblast-Induced Collagen Gel Contraction Using a Dynamic Microscale Platform. Front. Bioeng. Biotechnol..

[44] Clegg P.D., Strassburg S., Smith R.K. (2007). Cell phenotypic variation in normal and damaged tendons. Int. J. Exp. Pathol..

[45] Patterson-Kane J.C., Firth E.C., Hodgson D.R., McKeever K.H., McGowan C.M. (2014). Tendon, Ligament, Bone, and Cartilage: Anatomy, Physiology, and Adaptations to Exercise and Training. The Athletic Horse.

[46] Dos Anjos E.H.M., Mello M.L.S., Vidal B.D.C. Birefringence optical retardations in sections of rat calcaneal tendons cultured in vitro. Repository of Research Data of Unicamp (REDU).

[47] Valdetaro G.P., Aldrovani M., Padua I.R.M., Cristovam P.C., Gomes J.A.P., Laus J.L. (2016). Supra-organization and optical anisotropies of the extracellular matrix in the amniotic membrane and limbal stroma before and after explant culture. Biomed. Opt. Express.

[48] Lucke L.D., Bortolazzo F.O., Theodoro V., Fujii L., Bombeiro A.L., Felonato M., Dalia R.A., Carneiro G.D., Cartarozzi L.P., Vicente C.P. (2019). Low-level laser and adipose-derived stem cells altered remodelling genes expression and improved collagen reorganization during tendon repair. Cell Prolif..

[49] García J.R., Garcia A.J. (2014). Sensing rigidity. Nat. Mater..

[50] Yamamoto N., Ohno K., Hayashi K., Kuriyama H., Yasuda K., Kaneda K. (1993). Effects of Stress Shielding on the Mechanical Properties of Rabbit Patellar Tendon. J. Biomech. Eng..

[51] Wu S.Y., Kim W., Kremen T.J.J. (2022). In Vitro Cellular Strain Models of Tendon Biology and Tenogenic Differentiation. Front. Bioeng. Biotechnol..

[52] Cadby J.A., Buehler E., Godbout C., Van Weeren P.R., Snedeker J.G. (2014). Differences between the Cell Populations from the Peritenon and the Tendon Core with Regard to Their Potential Implication in Tendon Repair. PLoS ONE.

[53] Schwarz S., Gögel C., Ondruschka B., Hammer N., Kohl B., Schulze-Tanzil G. (2019). Migrating myofibroblastic iliotibial band-derived fibroblasts represent a promising cell source for ligament reconstruction. Int. J. Mol. Sci..

[54] Wall M.E., Banes A.J. (2005). Early responses to mechanical load in tendon: Role for calcium signaling, gap junctions and intercellular communication. J. Musculoskelet. Neuronal Interact..

[55] Wall M.E., Dyment N.A., Bodle J., Volmer J., Loboa E., Cederlund A., Fox A.M., Banes A.J. (2016). Cell Signaling in Tenocytes: Response to Load and Ligands in Health and Disease. Adv. Exp. Med. Biol..

[56] Schulze-Tanzil G., Mobasheri A., Clegg P.D., Sendzik J., John T., Shakibaei M. (2004). Cultivation of human tenocytes in high-density culture. Histochem. Cell Biol..

[57] Girke G., Kohl B., Busch C., John T., Godkin O., Ertel W., Schulze-Tanzil G. (2014). Tenocyte activation and regulation of complement factors in response to in vitro cell injury. Mol. Immunol..

[58] Frisch S.M., Vuori K., Ruoslahti E., Chan-Hui P.Y. (1996). Control of adhesion-dependent cell survival by focal adhesion kinase. J. Cell Biol..

[59] Ruoslahti E. (1997). Stretching Is Good for a Cell. Science.

[60] Chicurel M.E., Chen C.S., Ingber D.E. (1998). Cellular control lies in the balance of forces. Curr. Opin. Cell Biol..

[61] Tibbles L.A., Woodgett J.R. (1999). The stress-activated protein kinase pathways. Cell. Mol. Life Sci..

[62] Ramirez F., Rifkin D.B. (2003). Cell signaling events: A view from the matrix. Matrix Biol..

[63] Kjaer M. (2004). Role of Extracellular Matrix in Adaptation of Tendon and Skeletal Muscle to Mechanical Loading. Physiol. Rev..

[64] Jelinsky S.A., Li L., Ellis D., Archambault J., Li J., Andre M.S., Morris C., Seeherman H. (2011). Treatment with rhBMP12 or rhBMP13 increase the rate and the quality of rat Achilles tendon repair. J. Orthop. Res..

[65] Chang H.-N., Pang J.-H.S., Chen C.P., Ko P.-C., Lin M.-S., Tsai W.-C., Yang Y.-M. (2011). The effect of aging on migration, proliferation, and collagen expression of tenocytes in response to ciprofloxacin. J. Orthop. Res..

[66] Tokunaga T., Shukunami C., Okamoto N., Taniwaki T., Oka K., Sakamoto H., Ide J., Mizuta H., Hiraki Y. (2015). FGF-2 Stimulates the Growth of Tenogenic Progenitor Cells to Facilitate the Generation of *Tenomodulin*-Positive Tenocytes in a Rat Rotator Cuff Healing Model. Am. J. Sports Med..

