# Supplementary material for: High-Performance Polarization Microscopy Reveals Structural Remodeling in Rat Calcaneal Tendons Cultivated In Vitro
Source: Cells. 2023 Feb 10;12(4):566. doi: 10.3390/cells12040566 (PMC9953949; doi:10.3390/cells12040566)
Supplement: Supplementary file 1 [file cells-12-00566-s001.zip › cells-2106454-supplementary.pdf]

High-performance polarization microscopy reveals structural remodeling in rat calcaneal tendons cultivated *in vitro*

E.H.M. dos Anjos, M.L.S. Mello, B. de Campos Vidal

Dept. of Structural and Functional Biology, Institute of Biology, University of Campinas (Unicamp), 13083-862 Campinas, SP, Brazil

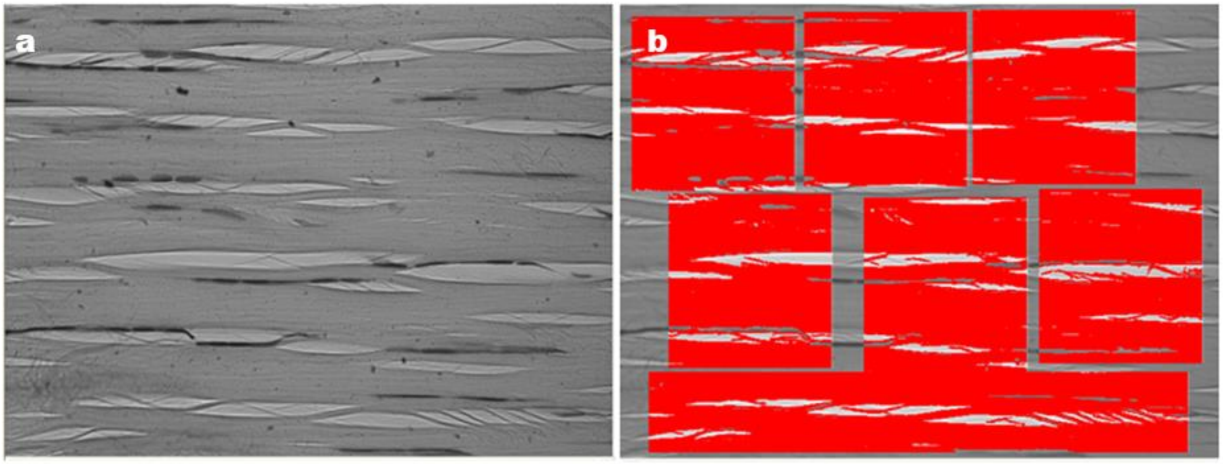

Supplementary Fig. 1. A large area of a tendon section after birefringence compensation (a) was segmented into several sub-areas (b) for more adequately comprising the variability of gray values in pixels that is a function of the inhomogeneous distribution of birefringent points. The scale bar equals 50 µm.

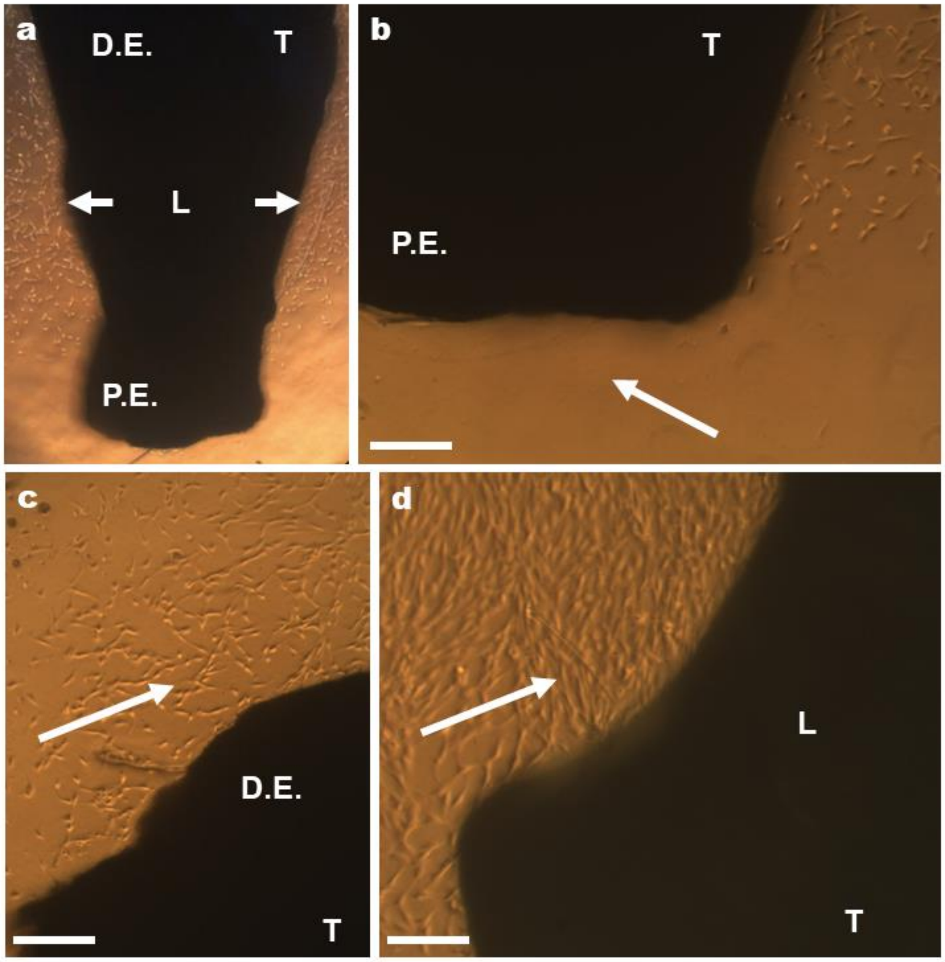

Supplementary Fig. 2. Tenocyte distribution in a rat tendon (T) at its 8-day-culture on plastic surface. Lateral tendon region (L) and distal (D.E.) and proximal (P.E.) tendon zones with respect to the original enthesis are shown. While no cell migration from the tendon is revealed at the P.E. zone (a, b – large arrow), sparse cell migration at D.E. (c – arrow), and concentrated cell migration close to L (d – arrow) are evident. The scale bars equal 250 μm.
